# The Views and Experiences of Families and Registered Nurses Regarding Hospital Care and Support of Children With Intellectual Disabilities: A Systematic Review

**DOI:** 10.1111/jar.70296

**Published:** 2026-08-19

**Authors:** Amirah Gadi, Lynne Marsh, Michael Brown

**Affiliations:** ^1^ School of Nursing and Midwifery Queen's University Belfast Belfast UK; ^2^ College of Nursing and Health Sciences Jazan University Jazan Saudi Arabia

**Keywords:** children, family‐centred care, hospital care, intellectual disability, nursing education, nursing practice

## Abstract

**Background:**

Children with intellectual disabilities experience poorer health outcomes and persistent inequities in hospital care. No previous review has integrated perspectives of families, registered nurses (RNs), nursing students and nurse educators to examine how family partnerships, workforce preparation and clinical practice intersect. This review synthesises evidence on hospital care and support for children with intellectual disabilities.

**Methods:**

Following PRISMA guidelines, four databases (CINAHL, MEDLINE, APA PsycINFO and Embase) were searched from inception to October 2025. Study quality was appraised using the MMAT.

**Results:**

Twenty‐eight studies were included, generating five themes: the significance of communication, partnership and family empowerment, environmental and systemic barriers, the role of education and professional development and emotional and psychological well‐being.

**Conclusions:**

Improving hospital care requires effective communication, meaningful family partnerships, supportive environments and reasonable adjustments, greater professional preparedness and emotional support. No eligible studies explored nursing students' or nurse educators' perspectives, revealing an evidence gap.

## Summary

- Children with intellectual disabilities often experience challenges in hospital care, including communication difficulties, limited reasonable adjustments and inconsistent support.
- Parents frequently take on the role of advocate and safety monitor for their child. While involvement is important, many families describe feeling tired, stressed or not fully listened to.
- Registered nurses report wanting to provide high‐quality care but often feel underprepared due to limited education and training in intellectual disability.
- There is very little research on how nursing students and nurse educators are prepared to support this group. Strengthening education and hospital systems could improve safety, inclusion and overall care experiences for children with intellectual disabilities and their families.

## 1 Introduction

The care of children with intellectual disabilities in hospital settings represents a critical challenge within contemporary healthcare. Intellectual disability, as defined by the World Health Organisation (WHO), refers to significant limitations in cognitive, communication, social and adaptive functioning, which can affect engagement with standard healthcare processes (WHO 2019). Compared with their peers, children with intellectual disabilities experience markedly poorer physical and mental health outcomes and are more likely to require frequent and prolonged hospital admissions (Hughes‐McCormack et al. 2020; Liao et al. 2021; Totsika et al. 2022). In recognition of these heightened needs, Articles 23 and 24 of the United Nations Convention on the Rights of the Child affirm their entitlement to dignity, appropriate support, access to specialised care and education and the highest attainable standard of health (UNICEF 2023). Nonetheless, persistent inequities in the quality and safety of hospital care remain, often driven by systemic barriers, stigma and inadequate service responsiveness (Mimmo, Harrison, et al. 2022; WHO 2023).

Family‐centred care (FCC) is widely recognised as a key approach to paediatric healthcare. It emphasises collaborative partnerships between healthcare professionals (HCPs), children and families, with families actively involved in care planning, decision‐making and care delivery (Mestre et al. 2024). This approach is particularly important for children with intellectual disabilities who may require additional support to communicate their needs and preferences. Evidence indicates that families continue to report inconsistent involvement in care planning and unaddressed healthcare needs, while simultaneously being relied upon to undertake multiple informal roles within hospital settings, including monitoring care, advocating for safety and mediating communication (Mimmo et al. 2019; Nicholson et al. 2022).

Registered nurses are central to hospital care for children with intellectual disabilities and their families, yet recent studies indicate persistent gaps in preparedness, particularly in identifying children with intellectual disabilities on admission and adapting communication and care practices in these settings (Mimmo, Hodgins, et al. 2022; Ong et al. 2022). These challenges are compounded by stigmatising assumptions about quality of life, which have been linked to inadequate education and training (Desroches et al. 2022; Noronha and Pawlyn 2023).

Similar concerns are reported among nursing students, whose confidence and attitudes remain constrained by limited curricular coverage and clinical exposure to intellectual disability care (Rozani et al. 2024; Warshawski 2025). Structured education and supervised experiential learning have shown promise in improving students' preparedness and attitudes (Edwards et al. 2022). However, this evidence has largely originated from educational settings rather than studies examining hospital care experiences and the extent to which nursing students' perspectives have been explored within hospital‐based research remains unclear.

Nurse educators play a critical role in shaping how intellectual disability is conceptualised and addressed within nursing education. Limited research suggests that disability content remains restricted and predominantly framed through a medical model, with insufficient attention to the biopsychosocial model and inclusive care practices, potentially contributing to gaps in graduate preparedness and ongoing inequities in care delivery and patient safety (Edwards and Hekel 2021; Lyon and Houser 2018; Moodley and McHunu 2022). The literature also highlights a disconnect between educators' expectations and students' perceived readiness, suggesting a need for more disability‐inclusive nursing education (Leonardsen et al. 2021). To date, evidence relating specifically to nurse educators' perspectives within the context of hospital care for children with intellectual disabilities remains limited.

Although hospital care experiences have been explored from the perspectives of families and HCPs (Mimmo et al. 2019; Ong et al. 2022), existing reviews have largely examined these groups in isolation. No previous synthesis has considered families, RNs, nursing students and nurse educators together, limiting understanding of how family partnership, clinical practice and workforce preparation collectively influence hospital care for children with intellectual disabilities. This review therefore aimed to synthesise evidence on the views and experiences of families, RNs, nursing students and nurse educators regarding the care and support of children with intellectual disabilities in hospital settings.

The review addressed the following question:

1. What are the views and experiences of families, RNs, nursing students and nurse educators regarding the care and support of children with intellectual disabilities in hospital settings?

## 2 Methods

A systematic review comprising qualitative, quantitative and mixed methods studies was undertaken to support a more comprehensive understanding of this complex area of practice (Stern et al. 2020). The review was reported in accordance with the Preferred Reporting Items for Systematic Reviews and Meta‐Analyses (PRISMA) 2020 reporting guidelines (Page et al. 2021) (Appendix A). Ethical approval was not required for this review as it involved the synthesis of data from previously published studies and did not involve the recruitment of human participants. A formal protocol was developed prior to conducting the review but was not registered in PROSPERO, OSF or another registry.

### 2.1 Eligibility Criteria

Inclusion and exclusion criteria were defined a priori to guide study selection (Morgan 2023). The eligibility criteria and search strategy were developed using the SPIDER tool (Sample, Phenomenon of Interest, Design, Evaluation, Research type), which is considered appropriate for qualitative and mixed methods evidence focused on views and experiences (Cooke et al. 2012). For the purposes of this review, the term ‘children’ was used to encompass children, adolescents and young people up to 24 years of age, in accordance with WHO definitions (WHO 2021). This age range was selected to reflect the inclusion of young people who may continue to receive paediatric services beyond traditional age boundaries. Studies including wider age ranges were eligible only if data relating to participants aged ≤ 24 years could be clearly identified and extracted separately from adult data. The SPIDER framework and associated eligibility criteria are presented in Table 1.

**TABLE 1:** SPIDER framework and eligibility criteria.

SPIDER components	Inclusion criteria	Exclusion criteria
Sample (S)	Families of children with intellectual disabilities; RNs; nursing students; nurse educators.	Samples not involving families, RNs, nursing students or nurse educators; studies focused solely on other professionals.
Phenomenon of Interest (PI)	Care and support of children (up to 24 years of age) with intellectual disabilities within hospital settings.	Studies not focused on intellectual disability (e.g., autism without co‐occurring intellectual disability); studies focused on adults; non‐hospital settings (e.g., community, schools, developmental centres); mental health inpatient, end‐of‐life or behaviour‐management‐only contexts.
Design (D)	Qualitative study designs (e.g., interviews, focus groups, ethnography); quantitative non‐interventional designs (e.g., surveys); mixed methods designs.	Non‐empirical designs; interventional or experimental studies; studies reporting clinical outcomes only; prevalence studies only.
Evaluation (E)	Views, experiences, perceptions and reported practices relating to hospital care and support.	Studies not reporting views, experiences or perspectives.
Research type (R)	Peer‐reviewed qualitative, quantitative and mixed methods studies published in English, with no date restrictions.	Secondary literature (e.g., dissertations, conference abstracts, editorials, book chapters and reviews).

### 2.2 Search Strategy

A preliminary search was conducted to identify relevant keywords, synonyms and subject headings using Google Scholar, database thesauri (e.g., MeSH) and databases including CINAHL Complete and MEDLINE to inform refinement of the final search strategy. A comprehensive search was subsequently conducted across four electronic databases: CINAHL Complete, MEDLINE, APA PsycINFO and Embase. The main search was conducted up to October 2025. Search terms included variations of: famil*, nurs*, nurs* educator*, nurs* education, child*, young people*, intellectual disab* and hospital*, alongside database‐specific subject headings and synonyms. Boolean operators (OR/AND), truncation and database‐specific limiters were applied to optimise sensitivity and relevance. The search strategy was developed and reviewed in collaboration with a specialist nursing librarian to enhance completeness and accuracy. Table 2 presents an example of the search strategy used in one of the electronic databases. The full search strategy is provided in Appendix B.

**TABLE 2:** CINAHL complete search strategy and results.

Database	No.	Index and keywords	No. of results
CINAHL complete	S1	‘nurs*’ OR (MH ‘Parents’) OR ‘famil*’ OR ‘carer*’ OR (MH ‘Caregivers’) OR ‘nurs* educat*’	1,556,923
	S2	‘Intellectual disab*’ OR ‘Learning disab*’ OR ‘developmental disab*’ OR ‘mental deficiency’ OR ‘mental* retard*’ OR ‘intellectual developmental disorder’ OR ‘intellectual developmental disability’ OR (MH ‘Persons with Intellectual Disabilities’) OR ‘cognitive disab*’ OR ‘intellectual impairment’ OR ‘mentally defective’ OR ‘psychosocial retard*’	54,700
	S3	‘child*’ OR (MH ‘Young Adult’) OR ‘young people’ OR ‘adolescent*’ OR ‘paediatric*’	1,407,232
	S4	(MH ‘Hospitals’) OR (MH ‘Hospitals, Paediatric’) OR (MH ‘Acute Care’) OR (MH ‘Emergency Service’) OR (MH ‘Emergency Room Visits’) OR (MH ‘Patient Admission’) OR (MH ‘Inpatients’) OR ‘in‐hospital’ OR (MH ‘Hospitalisation’) OR (MH ‘Paediatric Care’) OR (MH ‘Paediatric Units+’) OR (MH ‘Child Health Services’) OR ED	426,770
	S5	(S1 AND S2 AND S3 AND S4) Limiters: Published Date ≤ 31 October 2025	504
	S6	(S1 AND S2 AND S3 AND S4) Limiters: English language	498

### 2.3 Selection Process

All records were exported and screened in two stages. First, titles and abstracts were screened independently by two reviewers (A.G. and L.M.) for potential relevance. Second, the full texts of potentially eligible studies were retrieved and assessed independently by the same reviewers against the eligibility criteria (Cajal et al. 2020). Disagreements at either stage were resolved through discussion, with a third reviewer (M.B.) consulted when required. The review team met regularly to ensure consistency in the application of the eligibility criteria.

### 2.4 Quality Appraisal and Assessment

The methodological quality of the included studies was appraised using the Mixed Methods Appraisal Tool (MMAT) (Hong et al. 2018), which supports appraisal across qualitative, quantitative and mixed methods designs. MMAT responses were recorded as ‘Yes’, ‘No’ or ‘Cannot tell’ according to the clarity of reporting and evidence provided in each paper. Quality appraisal was undertaken to identify the methodological strengths and limitations of the included studies and to support interpretation of the review findings. Appraisal was led by the primary reviewer (A.G.) and a 10% sample was checked by the review team (L.M.) and (M.B.) to support consistency and reduce bias.

### 2.5 Data Extraction

Data were extracted from all included studies (*n* = 28) using a structured Microsoft Excel form developed for this review. Extracted data included: (i) citation details (authors, year, country), (ii) study aims, (iii) participant characteristics, (iv) methods, (v) main findings and (vi) recommendations. Where multiple publications appeared to report findings from the same underlying study, participant overlap was assessed during data extraction to avoid double counting in overall sample calculations. Data extraction was completed by the first reviewer (A.G.) and reviewed by the review team members to minimise errors and support rigour (Aromataris et al. 2014). Any uncertainties were discussed within the review team until consensus was reached. Table 3 presents a shortened version of the data extraction table. The full data extraction table is provided in Appendix C.

**TABLE 3:** Data extraction table.

No	Study citation	Aim	Participants	Methods	Main findings
1	Aston et al. (2014a) Canada	To explore the hospital care experiences of children with intellectual disabilities, parents and RNs.	Mothers (*n* = 17), RNs (*n* = 12) and children (*n* = 8)	Qualitative interviews	Parents and RNs described tensions around communication, parental roles and RNs' preparedness in caring for children with intellectual disabilities.
2	Aston et al. (2014b) Canada	To explore the personal, social and organisational experiences of children with intellectual disabilities, their parents and RNs in hospital settings.	Mothers (*n* = 17), RNs (*n* = 12) and children (*n* = 8)	Qualitative interviews	Relationships supported positive hospital experiences, while communication difficulties, stigma and organisational pressures undermined emotional and relational care.
3	Avis and Reardon (2008) UK	To explore parents' experiences of nursing care for children with intellectual disabilities in hospital settings.	Parents (*n* = 12)	Qualitative interviews	Parents reported emotional burden, ineffective communication and difficulties establishing trusting relationships with RNs.
4	Aydın et al. (2023) Turkey	To explore the challenges experienced by RNs when caring for children with intellectual disabilities in acute care settings.	RNs (*n* = 94)	Cross‐sectional survey	RNs reported communication, behavioural and organisational challenges alongside limited preparedness and support.
5	Bates et al. (2020) UK	To explore perceptions of treatment, accessibility and involvement in decision‐making within specialist cleft services for children with intellectual disabilities.	Children (*n* = 5), parents (*n* = 9) and HCPs (*n* = 9; RNs = 2)	Qualitative interviews	Parents reported distress during procedures, limited involvement in decision‐making and inadequate staff understanding of intellectual disability.
6	Breau et al. (2018) Canada	To explore the hospital experiences of children with intellectual disabilities and their families.	Mothers (*n* = 17), RNs (*n* = 12) and children (*n* = 8)	Qualitative interviews	Parents and RNs reported inadequate education and training, contributing to low confidence, communication difficulties and reliance on informal learning.
7	Brown and Guvenir (2009) UK	To explore the experiences of children with intellectual disabilities, families and RNs during hospital admission.	Carers (*n* = 13), RNs (*n* = 13) and children (*n* = 2)	Qualitative interviews	Families and RNs reported anxiety, staff under preparedness and reliance on carers for communication and behavioural support, while individual rooms helped reduce distress.
8	Carter et al. (2016) UK	To explore HCPs' experiences of assessing and managing pain in children with profound cognitive impairments.	HCPs (*n* = 19; RNs = 8)	Qualitative interviews	Communication barriers complicated pain assessment, leading RNs to rely on parents' expertise to support individualised pain management.
9	Cashin et al. (2022) Australia	To compare RNs' perceived preparedness, knowledge, confidence and comfort in caring for individuals with intellectual disabilities and/or autism spectrum disorder across different healthcare settings.	RNs (*n* = 639)	Cross‐sectional survey	Paediatric RNs reported greater confidence in caring for children with intellectual disabilities than ICU and ED RNs but identified ongoing education and training needs.
10	Dinsmore (2012) UK	To explore the hospital experiences of children and young people with intellectual disabilities and their caregivers.	Carers (*n* = 10), adults with intellectual disability (*n* = 5)	Qualitative interviews	Gaps in specialist intellectual disability support, communication, care planning and post‐discharge support affected hospital experiences.
11	Downs et al. (2016) Australia	To explore family experiences and satisfaction with hospital care following spinal fusion surgery in girls with Rett syndrome.	Families (*n* = 392)	Questionnaire (closed‐ and open‐ended questions)	Families reported positive experiences associated with specialist expertise, effective communication and recognition of parental expertise, but inconsistent care and delays caused distress.
12	Iversen et al. (2013) Norway	To explore parents' experiences of the surgical care received by their child with cerebral palsy.	Parents (*n* = 12)	Qualitative interviews	Parents reported emotional distress and vulnerability, with trust in HCPs dependent on effective communication, recognition of parental expertise and collaborative care.
13	Jensen et al. (2025) USA	To explore the healthcare experiences of children with medical complexities and their caregivers, including the impact of the COVID‐19 pandemic.	Caregivers (*n* = 175)	Mixed methods study	Families reported distress associated with repeated interventions, fragmented services and poor care coordination.
14	Kenten et al. (2019) UK	To examine hospital approaches to identifying children with intellectual disabilities.	HCPs (*n* = 2326; 1020 RNs)	Mixed methods study	RNs viewed identification systems as important for safe, individualised care but reported inconsistent practices, communication barriers and training needs.
15	Lamptey (2022) Ghana	To explore families' experiences of navigating the healthcare system for children with intellectual and developmental disabilities.	Caregivers (*n* = 22)	Qualitative interviews	Families reported service barriers, communication challenges and fragmented care coordination that limited responsiveness to children's needs.
16	Lewis et al. (2019) Australia	To explore RNs' experiences of providing care for children with intellectual disabilities in acute paediatric settings.	RNs (*n* = 8)	Qualitative interviews	Communication barriers and complex care needs increased nursing workload, while familiar routines and strong nurse‐parent partnerships supported personalised care.
17	Mimmo, Hodgins, et al. (2022) Australia	To explore RNs' perspectives on positive care experiences for inpatient children with intellectual disabilities.	RNs (*n* = 29)	Qualitative focus groups	RNs described quality care as dependent on communication, trust, understanding individual needs and collaboration with parents, despite time and resource constraints.
18	Moloney et al. (2023) Ireland	To explore parents' experiences of reasonable adjustments in acute care settings for individuals with intellectual disabilities.	Parents (*n* = 6)	Qualitative interviews	Parents reported that limited reasonable adjustments, inadequate sensory‐friendly environments and advocacy burdens negatively affected care experiences.
19	Murdoch and Chang (2022) UK	To explore parents' experiences of procedural anxiety and hospital care for children with intellectual disabilities.	Parents (*n* = 6)	Qualitative interviews	Parents experienced emotional distress and isolation, with advocacy challenges, limited specialist support and inconsistent staff expertise contributing to anxiety.
20	Ong et al. (2024) Australia	To examine patient safety concerns for children with intellectual disabilities and inform improvements to patient safety frameworks.	Parents (*n* = 13)	Qualitative interviews	Parents reported that poor communication, dismissive attitudes and limited adjustments undermined safety and increased caregiving burden.
21	Oulton et al. (2015) UK	To explore perspectives on the hospital care needs of children with intellectual disabilities and their families.	HCPs (*n* = 27; 10 RNs)	Ethnographic approach	Individualised care depended on staff expertise and parent collaboration, although knowledge and communication gaps increased reliance on families.
22	Oulton et al. (2018) UK	To explore the needs and experiences of children with intellectual disabilities during hospitalisation.	Children (*n* = 9) and at least one of their parents.	Ethnographic approach	Children valued personalised care, communication, choice and familiar routines, which helped reduce anxiety and improve hospital experiences.
23	Oulton et al. (2019) UK	To explore organisational influences on healthcare delivery and staff perceptions of caring for children with intellectual disabilities. To examine dedicated intellectual disability nursing services in children's hospitals and compare staff perspectives across hospitals with and without these services	HCPs (*n* = 1729; 850 RNs)	Mixed methods study	Limited specialist support, communication challenges and inconsistent organisational support constrained RNs' ability to provide individualised care.
24	Oulton et al. (2020) UK	To explore parents' experiences and expectations of partnerships with HCPs during hospital care for children with intellectual disabilities.	Parents (*n* = 9)	Ethnographic approach	Positive care experiences were supported by trusting partnerships, effective communication and respect for parental expertise.
25	Oulton et al. (2022) UK	To identify organisational and individual factors influencing equitable access to hospital care for children with intellectual disabilities and their families.	HCPs (*n* = 2853; 1109 RNs; number of RNs in specific phases was not specified) Children (*n* = 1419; 198 with intellectual disability) Families (*n* = 1371; number of parents of children with intellectual disability was not specified)	Mixed methods case study	Limited specialist support, training and environmental adjustments hindered individualised care for children with intellectual disabilities.
26	Oulton et al. (2024) UK	To explore children's and parents' perspectives on the emotional and physical effects of hospitalisation for children with intellectual disabilities.	Parents (*n* = 52) and children (*n* = 42)	Mixed methods case study	Hospital experiences were shaped by disrupted routines, sensory sensitivities, procedural anxiety and communication barriers affecting pain management.
27	Oulton et al. (2025) UK	To explore stakeholder perspectives on patient safety for children with intellectual disabilities during inpatient hospital care.	Parents (*n* = 52) and HCPs (*n* = 98; number of RNs not specified)	Qualitative interviews	Parents and RNs reported safety concerns related to communication barriers, variable staff expertise and inadequately adapted care environments.
28	Seliner et al. (2016) Switzerland	To examine parental burden, family‐centred care and quality of life among children with profound intellectual disabilities and their families.	Mothers (*n* = 98) and fathers (*n* = 19)	Mixed methods study	The findings highlighted the importance of family‐centred care in supporting children and families during hospitalisation.

### 2.6 Data Analysis and Integration

A convergent integrated approach was used to synthesise qualitative and quantitative evidence within a single analytic framework (Stern et al. 2020). Quantitative findings from quantitative descriptive studies and mixed methods studies were transformed into narrative statements (‘qualitised’) to enable integration with qualitative findings. All extracted findings were then synthesised using reflexive thematic analysis (RTA) by Braun and Clarke (2022), selected due to the predominance of qualitative evidence and the review aim of identifying patterns in views and experiences across heterogeneous study designs. Initial coding was undertaken inductively by the first author (A.G.), with codes subsequently organised into candidate themes. Theme development and refinement involved ongoing discussion with the review team (L.M. and M.B.) until a coherent thematic framework was developed. Consistent with RTA, researcher interpretation was recognised as an active part of the analytic process. Transparency and credibility were supported through regular team discussions, documentation of analytic decisions and iterative review of themes throughout the synthesis. A meta‐analysis was not undertaken because of substantial heterogeneity in study designs, participant groups, outcome measures and the predominance of qualitative evidence, making quantitative pooling inappropriate.

## 3 Results

### 3.1 Search Outcomes

**FIGURE 1:**
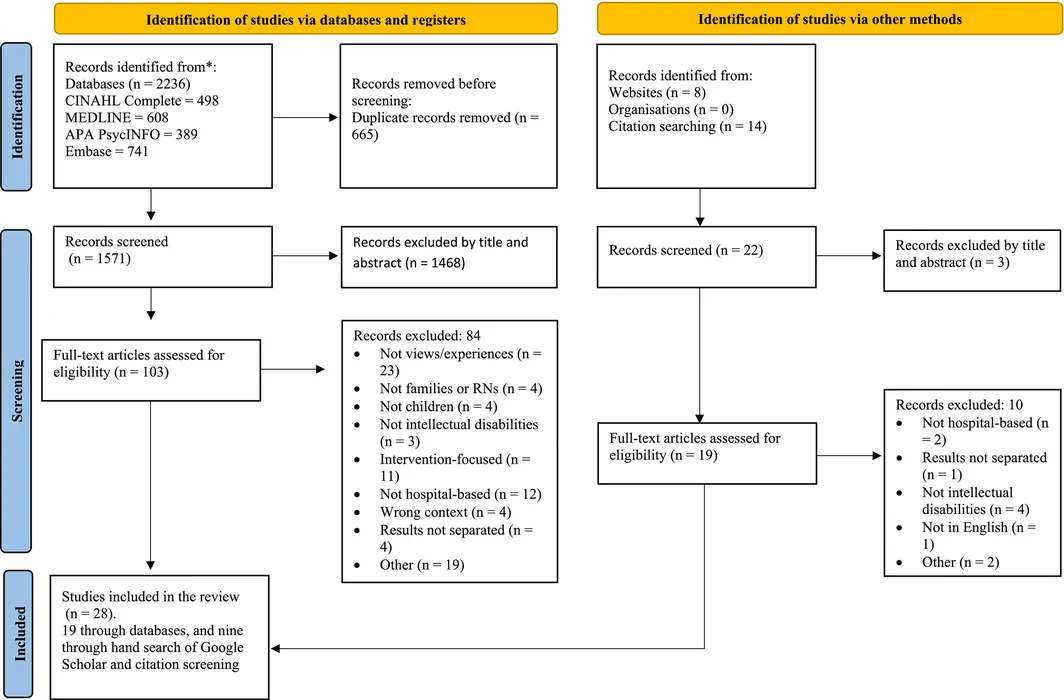
PRISMA flow diagram.

A total of 2236 records were identified across all databases and imported into EndNote version 21. After the removal of duplicates (*n* = 665), 1571 records remained for title and abstract screening, of which 1468 were excluded. Consequently, 103 full‐text articles were assessed for eligibility and 84 were excluded. Citation searching and hand‐searching of reference lists identified a further 22 records; three were excluded following title and abstract screening and 19 underwent full‐text assessment, of which 10 were excluded. In total, 28 studies met the eligibility criteria and were included in the review (Figure 1).

### 3.2 Quality Ratings

Quality appraisal using the MMAT indicated that the included studies were generally of acceptable methodological quality. Common strengths included clearly stated research questions, appropriate study designs and coherence between study aims, data collection and analysis. Across qualitative studies, common limitations included insufficient detail regarding data collection procedures, analytic transparency and researcher reflexivity (Aston et al. 2014b; Brown and Guvenir 2009; Dinsmore 2012; Lewis et al. 2019). Quantitative studies demonstrated minor limitations related to sample size, response bias or incomplete reporting of participant characteristics (Aydın et al. 2023; Cashin et al. 2022). Mixed methods studies most frequently lacked clarity regarding the integration of qualitative and quantitative components or the reporting of methodological rigour in the qualitative phase (Downs et al. 2016; Oulton et al. 2024, 2019, 2022; Seliner et al. 2016). No studies were excluded on the basis of quality appraisal. Full appraisal results are presented in Appendix D.

### 3.3 Study and Participant Characteristics

The included body of evidence was methodologically diverse but predominantly qualitative in nature (*n* = 19), followed by mixed methods studies (*n* = 7) and quantitative cross‐sectional surveys (*n* = 2). Geographically, the literature was heavily weighted towards high‐income Western countries, particularly the United Kingdom (UK) (*n* = 14), Australia (*n* = 5) and Canada (*n* = 3). Additional studies were conducted in Norway (*n* = 1), the United States (USA) (*n* = 1), Ireland (*n* = 1), Switzerland (*n* = 1), Turkey (*n* = 1) and Ghana (*n* = 1). The total sample size across the included studies was 6223 participants. Study samples ranged from small qualitative interview studies (*n* = 6) to larger survey‐based studies (*n* = 198) for families and from individual interviews (*n* = 2) to large‐scale surveys (*n* = 984) for RNs. It should be noted that several publications arose from the same wider NIHR‐funded mixed‐methods study (Oulton et al. 2022). These publications were treated as linked reports from a single parent study rather than as independent studies to avoid double counting participants. Where publications reported distinct phases, stakeholder groups or analytic foci, their findings were extracted separately, but sample characteristics were counted once at parent‐study level. In addition, the exact number of RNs was not clearly disaggregated in all phases; therefore, only the total number of HCPs was reported (Oulton et al. 2022). Appendix E presents the distribution of study countries and populations.

Children were described as having intellectual and developmental disabilities, most commonly intellectual disability, global developmental delay and autism spectrum disorder, often associated with genetic syndromes and complex neurological, physical, sensory and multi‐system comorbidities, including cerebral palsy, epilepsy, congenital anomalies and communication impairments. Several studies did not clearly report or specify participants' diagnoses, limiting the precision with which clinical profiles could be compared across the evidence base (Avis and Reardon 2008; Brown and Guvenir 2009; Moloney et al. 2023; Oulton et al. 2022).

### 3.4 Data Synthesis Results

Findings from the 28 included studies were integrated using reflexive thematic analysis. Within each theme, family and RN perspectives are presented separately where possible to highlight areas of convergence and divergence. Five interrelated themes were identified, describing how hospital care for children with intellectual disabilities is experienced by families and RNs: (1) The significance of communication; (2) Partnership and family empowerment; (3) Environmental and systemic barriers; (4) The role of education and professional development; and (5) Emotional and psychological well‐being (Appendix F).

#### 3.4.1 The Significance of Communication

Communication emerged as a prominent theme across the included studies, shaping children's involvement in care and influencing family experiences, care continuity and perceptions of safety in hospital settings (Aston et al. 2014a, 2014b; Avis and Reardon 2008; Aydın et al. 2023; Bates et al. 2020; Breau et al. 2018; Brown and Guvenir 2009; Carter et al. 2016; Cashin et al. 2022; Dinsmore 2012; Downs et al. 2016; Iversen et al. 2013; Jensen et al. 2025; Kenten et al. 2019; Lamptey 2022; Lewis et al. 2019; Mimmo, Hodgins, et al. 2022; Moloney et al. 2023; Ong et al. 2024; Oulton et al. 2024, 2025, 2018, 2020, 2015, 2019, 2022).

Direct and meaningful interaction between HCPs and children was considered essential by many families, yet staff were often perceived to underestimate children's ability to understand and to communicate primarily through parents (Aston et al. 2014b; Avis and Reardon 2008). This was described as marginalising children's voices, reinforcing low expectations and weakening trust and relational connection. Communication difficulties were also evident at team and service levels, with families often describing the need to bridge gaps between professional groups by repeating information and supporting continuity during transitions (Mimmo, Hodgins, et al. 2022), particularly when documentation of children's priorities and needs was limited (Oulton et al. 2018).

Within nursing practice, communication challenges were linked to uncertainty and limited confidence in using augmentative and alternative communication (AAC) strategies, visual supports or tailored approaches to elicit children's preferences and histories (Aydın et al. 2023; Breau et al. 2018). Registered nurses also described frustration and ethical concerns where they felt unable to engage children effectively or ensure that children were genuinely involved in their care (Aston et al. 2014b; Breau et al. 2018).

Family experiences suggested that communication was valued when HCPs actively engaged with children with intellectual disabilities, avoided patronising language, responded to individual needs and shared information with parents in a timely and collaborative manner. These practices were perceived to support care continuity, trust and safety, particularly during transitions (Ong et al. 2024; Oulton et al. 2020). In practice, these communicative ideals were not always realised, with tensions emerging around disclosure and information‐seeking. Some RNs hesitated to enquire about a child's intellectual disability due to parental concerns about stigma, labelling or the risk of unequal treatment (Kenten et al. 2019).

Despite differences in experience, families and RNs consistently highlighted the value of tailored communication methods and accessible resources, including visual timetables, social stories, easy‐read materials and hospital passports, in supporting communication with children with intellectual disabilities. However, both groups reported that implementation remained inconsistent because of time pressures, variable uptake and practical barriers, such as language differences (Kenten et al. 2019; Moloney et al. 2023; Ong et al. 2024; Oulton et al. 2022). From an RN perspective, training in communication approaches, such as Makaton, was viewed as beneficial, although maintaining competence required regular practice, which was often difficult within busy ward settings (Oulton et al. 2015, 2019).

#### 3.4.2 Partnership and Family Empowerment

Experiences of partnership and empowerment were closely linked to the extent to which families' expertise and advocacy were recognised and meaningfully incorporated into decision‐making and everyday care practices (Aston et al. 2014a, 2014b; Avis and Reardon 2008; Aydın et al. 2023; Bates et al. 2020; Breau et al. 2018; Brown and Guvenir 2009; Carter et al. 2016; Cashin et al. 2022; Dinsmore 2012; Downs et al. 2016; Iversen et al. 2013; Jensen et al. 2025; Kenten et al. 2019; Lewis et al. 2019; Mimmo, Hodgins, et al. 2022; Moloney et al. 2023; Murdoch and Chang 2022; Ong et al. 2024; Oulton et al. 2025, 2020, 2015, 2019, 2022; Seliner et al. 2016).

Families described persistent power imbalances and limited opportunities to influence decisions where professional authority outweighed parental knowledge (Bates et al. 2020; Dinsmore 2012; Jensen et al. 2025). Advocacy for reasonable adjustments was at times met with stigma, with some parents reporting that they were perceived as ‘difficult’ or ‘pushy’, which eroded trust and reduced confidence in raising concerns about care (Aston et al. 2014a; Moloney et al. 2023; Murdoch and Chang 2022).

Although RNs frequently acknowledged parents' unique knowledge of their child, they also described constraints that limited consistent partnership, including high workloads, time pressures, inadequate preparation for intellectual disability care and inconsistent organisational policies (Aydın et al. 2023; Breau et al. 2018; Oulton et al. 2022). Registered nurses also described tensions within partnership relationships, including role ambiguity and occasional perceptions that highly experienced parents challenged clinical decision‐making (Carter et al. 2016). In such circumstances, parents assumed responsibility for coordinating aspects of care, providing information, guiding staff and monitoring safety, particularly when they perceived risks or inconsistencies in care practices (Oulton et al. 2025).

For families, effective partnerships were characterised by emotional support, attentiveness to both the child's and family's needs, respect, empathy, trust and shared responsibility. These partnerships were strengthened when parents were recognised as credible contributors to care rather than informal substitutes for HCPs (Aston et al. 2014b; Iversen et al. 2013; Seliner et al. 2016). More positive partnership experiences were reported by both families and RNs when relationships developed over repeated interactions, staff actively listened to families and negotiated roles and children were engaged in ways that promoted familiarity and comfort (Lewis et al. 2019; Mimmo, Hodgins, et al. 2022; Oulton et al. 2020).

#### 3.4.3 Environmental and Systemic Barriers

Environmental and organisational factors consistently influenced whether hospital care could be delivered in an inclusive, flexible and individualised manner for children with intellectual disabilities (Aston et al. 2014a, 2014b; Avis and Reardon 2008; Aydın et al. 2023; Bates et al. 2020; Breau et al. 2018; Brown and Guvenir 2009; Dinsmore 2012; Downs et al. 2016; Jensen et al. 2025; Kenten et al. 2019; Lamptey 2022; Lewis et al. 2019; Mimmo, Hodgins, et al. 2022; Moloney et al. 2023; Murdoch and Chang 2022; Ong et al. 2024; Oulton et al. 2024, 2025, 2018, 2015, 2019, 2022).

Physical environments were described by families as poorly adapted to children's sensory, privacy and comfort needs, with limited access to quiet spaces, private rooms, appropriate toileting facilities, play resources and other reasonable adjustments (Moloney et al. 2023; Oulton et al. 2024). The absence of such adjustments contributed to heightened stress and anticipatory anxiety among families during hospital admissions, while RNs reported a reduced capacity to prevent escalation and support emotional regulation (Moloney et al. 2023; Oulton et al. 2025).

Across both stakeholder groups, system‐level constraints were recognised as barriers to equitable care, particularly the limited availability of tailored resources for children with intellectual disabilities. These included simplified communication tools, disability‐specific equipment and structured supports, with their absence undermining individualised care delivery (Ong et al. 2024; Oulton et al. 2025, 2022). The limited availability of specialist staff, such as intellectual disability‐trained nurses, play specialists and pain specialists, was also highlighted as a key systemic challenge (Aydın et al. 2023; Murdoch and Chang 2022; Oulton et al. 2022). While carers advocated for greater specialist input to support both families and generalist RNs (Dinsmore 2012), RNs reported that access to intellectual disability nurse support, though valued and associated with enhanced perceived capability, did not improve generalist RNs' confidence, perceptions of patient value or safety or access to appointments (Oulton et al. 2019).

A further systemic issue identified across RN accounts concerned the absence of consistent identification processes for children with intellectual disabilities (Aydın et al. 2023; Oulton et al. 2022). Identification or ‘flagging’ systems were viewed by RNs as potentially useful in prompting reasonable adjustments and reducing variability in care, although concerns regarding stigma and parental reluctance to disclose disability status persisted (Kenten et al. 2019). Broader pressures reported by families, including long waits, fragmented services, discharge delays and staffing constraints, were repeatedly linked to discontinuity, reduced responsiveness and the shifting of responsibility for safety onto parents (Dinsmore 2012; Lamptey 2022; Oulton et al. 2025).

#### 3.4.4 The Role of Education and Professional Development

Education and professional development emerged as critical determinants of HCPs' confidence, competence and preparedness when caring for children with intellectual disabilities in hospital settings (Aston et al. 2014a, 2014b; Aydın et al. 2023; Bates et al. 2020; Breau et al. 2018; Brown and Guvenir 2009; Carter et al. 2016; Cashin et al. 2022; Iversen et al. 2013; Kenten et al. 2019; Lamptey 2022; Mimmo, Hodgins, et al. 2022; Moloney et al. 2023; Murdoch and Chang 2022; Ong et al. 2024; Oulton et al. 2024, 2025, 2020, 2015, 2019, 2022).

Feeling of being underprepared to care for children with intellectual disabilities was commonly described by RNs, with gaps in formal education contributing to uncertainty and reliance on families to guide care (Aston et al. 2014a; Bates et al. 2020; Breau et al. 2018). In a large Australian study involving 639 RNs across various specialties, Cashin et al. (2022) identified significant shortcomings in nursing curricula, with paediatric RNs expressing a need for targeted education. Both families and RNs linked limited preparation to challenges in communication, recognising symptoms, managing unpredictable behaviours, pain management and supporting children through procedures (Aydın et al. 2023; Carter et al. 2016; Iversen et al. 2013; Oulton et al. 2024). In some accounts, parents described HCPs using restrictive or outdated approaches in response to behavioural distress, which intensified family anxiety and mistrust (Lamptey 2022).

There was broad agreement among families and RNs regarding the importance of practical, skills‐based learning focused on communication strategies, family‐centred practice and addressing stigma and attitudes (Breau et al. 2018; Ong et al. 2024). Registered nurses described experiential learning and supported clinical exposure as important for reducing fear and building confidence, particularly in communication, advocacy and pain management (Aston et al. 2014b; Aydın et al. 2023; Carter et al. 2016; Mimmo, Hodgins, et al. 2022). Both groups viewed the involvement of people with intellectual disabilities and families in educational initiatives as beneficial for challenging assumptions, strengthening understanding of lived experience and supporting more individualised care (Breau et al. 2018; Oulton et al. 2020, 2015). Registered nurses reported that input from intellectual disability nurse specialists supported skills development and confidence among generalist RNs, while shared learning approaches promoted longer‐term capacity building, particularly for newly qualified and less experienced staff (Mimmo, Hodgins, et al. 2022; Oulton et al. 2019).

#### 3.4.5 Emotional and Psychological Well‐Being

Emotional and psychological well‐being was woven throughout accounts from families and RNs, shaping perceptions of safety, trust and care quality (Aston et al. 2014a, 2014b; Avis and Reardon 2008; Bates et al. 2020; Breau et al. 2018; Brown and Guvenir 2009; Carter et al. 2016; Cashin et al. 2022; Downs et al. 2016; Iversen et al. 2013; Jensen et al. 2025; Lewis et al. 2019; Moloney et al. 2023; Murdoch and Chang 2022; Ong et al. 2024; Oulton et al. 2024, 2025, 2018, 2020, 2022; Seliner et al. 2016). Families emphasised that safety extended beyond physical outcomes to include emotional security, dignity and the minimisation of distress (Ong et al. 2024). Anxiety was frequently linked to repeated admissions, inconsistent care practices, limited emotional support and experiences of not being listened to (Avis and Reardon 2008; Downs et al. 2016; Oulton et al. 2022). Many also reported heightened distress during invasive or unfamiliar procedures, including restraint, cannulation and anaesthesia, often accompanied by feelings of guilt, helplessness and constant vigilance (Bates et al. 2020; Jensen et al. 2025; Murdoch and Chang 2022). Sensory overload within ward environments further intensified distress for children and families (Moloney et al. 2023; Oulton et al. 2024, 2018). Registered nurses also described emotional strain associated with uncertainty, limited confidence and perceived gaps in knowledge, particularly in areas such as pain assessment and responses to behavioural distress (Breau et al. 2018; Brown and Guvenir 2009; Carter et al. 2016). Although RNs recognised the importance of providing emotional support, time pressures, staffing constraints and competing priorities were reported to limit their capacity to deliver the level of relational care they valued and families required (Aston et al. 2014b). Registered nurses reported greater confidence and emotional comfort when specialist support and greater exposure to intellectual disability care were available, highlighting the influence of preparedness and the care environment on both staff experience and the emotional quality of care (Cashin et al. 2022).

## 4 Discussion

This review synthesised evidence on the views and experiences of families and RNs regarding hospital care and support for children with intellectual disabilities. Although the review aimed to include nursing students and nurse educators, no eligible studies were identified, highlighting a substantive gap in the evidence base. Across the included studies, challenges related to communication, partnership, environmental and system conditions, education and professional development and emotional and psychological well‐being were closely interconnected. Rather than reflecting isolated failures, the findings indicate that hospital care experiences are shaped by the interaction of relational practices, organisational conditions and workforce preparation.

Communication emerged as more than a technical skill, functioning as a relational and ethical foundation for inclusive care. Across the reviewed studies, communication difficulties occurred at multiple levels, ranging from discontinuities in documentation and information transfer (Mimmo, Hodgins, et al. 2022; Oulton et al. 2018) to limited RN confidence in adapting communication approaches (Aydın et al. 2023; Breau et al. 2018). A consistent pattern across the review was the marginalisation of children's voices, often justified by assumptions about capacity (Aston et al. 2014a). These findings align with wider literature suggesting that hospital environments prioritise task completion and throughput, leaving limited time for supported interaction and positioning parents as default communication intermediaries (Hemsley and Balandin 2014; Lewis et al. 2017; Sharkey et al. 2016).

Meaningful communication is not dependent on verbal fluency alone. Evidence on non‐verbal communication highlights the clinical and moral importance of attending to cues, recognising personhood and allowing time and intentionality to support participation (Penninga et al. 2025; Sato 2022). Within this context, playful interaction, humour and emotionally attuned engagement can be understood as indicators of quality, particularly when children are supported with visual resources and opportunities to express preferences (Mimmo, Hodgins, et al. 2022; Oulton et al. 2024).

The findings also point to a persistent implementation gap. Although hospital passports, visual schedules, Makaton and other supports are widely promoted, their use remains inconsistent (Mimmo, Hodgins, et al. 2022; Moloney et al. 2023; Oulton et al. 2015, 2022). Effective adaptation of these tools and communication skills depends on reinforcement through supervision, institutional leadership and integration into everyday workflows, rather than stand‐alone training sessions (Andzik and Chung 2021; Jackson et al. 2024; Marsh and Brown 2025; Uthoff et al. 2021; Ware et al. 2024). This has direct implications for nurse education, where communication competence with children with intellectual disabilities should be treated as a core clinical capability, supported through explicit teaching, practice opportunities and assessment.

Although FCC is widely endorsed in policy, the review indicates that partnership is often inconsistently enacted in practice. Families described exclusion from decision‐making, unclear role negotiation and stigmatising responses to advocacy when seeking adjustments or raising safety concerns (Bates et al. 2020; Murdoch and Chang 2022; Ong et al. 2024). This pattern aligns with the concept of epistemic injustice in healthcare, whereby family knowledge is discounted relative to professional authority, limiting shared decision‐making and eroding relational trust (King et al. 2025; Smith et al. 2015; Watts et al. 2014).

RNs' accounts further suggest that collaborative working is shaped by context as much as intention. High workloads and limited preparation in FCC principles restricted opportunities for meaningful collaboration (Aydın et al. 2023; Kenten et al. 2019; Oulton et al. 2022), contributing to role ambiguity and shifting responsibility onto parents for vigilance and continuity, particularly during admissions and care transitions (Brown et al. 2020; Coyne 2015; Flanagan et al. 2023; Oulton et al. 2025).

Wider evidence, including the *Learning from Lives and Deaths—People with a Learning Disability and Autistic People* (LeDeR) report, indicates that poor communication and weak partnership can contribute to avoidable harm, underscoring partnership as a safety issue as well as a relational (White et al. 2023). These findings highlight that effective FCC requires the deliberate development of relational competencies such as empathetic listening, collaborative planning, culturally responsive communication and respectful role negotiation. These must be supported through professional education and reinforced by ward leadership and organisational norms (Dewan et al. 2024; Jang 2020; Mimmo, Hodgins, et al. 2022; Wong et al. 2023), with emotionally attuned shared decision‐making shown to enhance family satisfaction and psychological well‐being (Mas et al. 2019; Mestre et al. 2024).

Environmental and system‐level conditions consistently shaped whether inclusive care could be achieved. Families and RNs described physical environments that were poorly adapted to children's sensory and behavioural needs, with limited access to quiet spaces and child‐friendly resources (Moloney et al. 2023; Oulton et al. 2022). Emerging intervention evidence demonstrates that proactively designed, sensory‐adapted environments can reduce stress and support inclusion, particularly when adjustments are planned in advance rather than improvised in response to crisis (Roos et al. 2024; Watchorn et al. 2025).

The review also identified wider systemic barriers, including limited access to intellectual disability‐specific equipment and communication tools, variable specialist support and inconsistent identification and flagging processes (Aydın et al. 2023; Kenten et al. 2019; Ong et al. 2024; Oulton et al. 2022). These findings underscore the need to reconceptualise reasonable adjustments in acute hospital settings as a system‐level responsibility rather than a series of ad hoc individual responses. As Moloney et al. (2025) argue, effective implementation requires attention to the interrelated influences of organisational context, service structures and care environments, highlighting the importance of examining how reasonable adjustments are embedded, sustained and operationalised within complex healthcare systems.

While specialist intellectual disability roles have the potential to support inclusive care and staff capability, their effectiveness depends on integration, clarity of remit and organisational commitment rather than symbolic presence alone (Bur et al. 2021; Mafuba et al. 2023; Oulton et al. 2019; Sheehan et al. 2024). Similarly, identification and flagging systems can facilitate early planning and improve access to adjustments, but only when implemented ethically and accompanied by safeguards against stigmatisation, ensuring that identification prompts action rather than labelling (Heslop et al. 2019; Moloney et al. 2021; Ong et al. 2024). Collectively, these findings highlight the need for a broader cultural shift in hospital practice, in which disability‐responsive design, early recognition of individual needs and proactive planning for reasonable adjustments are embedded systematically, alongside the meaningful involvement of disabled people in shaping inclusive hospital care.

Education and professional development emerged as both a central enabler of inclusive care and a persistent constraint. Registered nurses often described uncertainty attributed to limited undergraduate preparation and inadequate supported exposure to caring for children with intellectual disabilities (Cashin et al. 2022). Across the wider literature, intellectual disability content remains marginal within nursing curricula, with consequences for communication, behaviour support, pain assessment and confidence in complex care (Aydın et al. 2023; Carter et al. 2016; Ciccone et al. 2023; Howie et al. 2021; Spassiani et al. 2020; Tumanggor et al. 2024). Where formal preparation is weak, learning is displaced to the bedside and frequently onto families, reinforcing inequity by transferring responsibility to parents and contributing to variability in care quality.

The findings emphasise embedding intellectual disability as a core, longitudinal capability within paediatric nursing curricula rather than an optional add‐on. Experiential, reflective and simulation‐based approaches, alongside narrative and blended learning, show promise in strengthening competence, confidence and relational sensitivity, particularly in responding to distress or challenging situations (Cashin et al. 2023; Mitchell et al. 2020; Taggart et al. 2022; Truong et al. 2021; Watson et al. 2025). Co‐designed education with people with intellectual disabilities and families is consistently associated with reduced stigma, enhanced empathy and learning grounded in lived experience (Bates et al. 2020; Fisher et al. 2022; Oulton et al. 2020). Evidence of long‐term impact remains limited (Hay et al. 2024) and sustained benefit depends on reinforcement within practice environments, organisational investment and equitable access to supported placements and specialist mentorship (Doody et al. 2020; Furst and Salvador‐Carulla 2019).

Emotional and psychological well‐being emerged not merely as an additional theme but as a lens through which communication, partnership and systemic conditions could be understood. Families described anticipatory anxiety, moral distress and, in some cases, experiences consistent with medical trauma when exposed to restraint, invasive procedures or hospital environments that intensified sensory overload and could escalate into unsafe situations during hospital care (Jensen et al. 2025; Moloney et al. 2023; Murdoch and Chang 2022; Oulton et al. 2024). Evidence beyond this review indicates that emotional well‐being remains insufficiently prioritised within service responses to profound intellectual disability, with emerging safety literature suggesting that adverse events may arise not only from clinical error but also from unmet psychosocial needs and cumulative distress (Lahaije et al. 2023; Ong 2024).

Relationally attuned interactions and structured interventions, such as medical play, procedural preparation, familiar routines, sensory‐adapted environments and access to psychological support, have been shown to reduce distress and anxiety and to support emotional regulation (Chrisler et al. 2021; Inoue et al. 2021; Mimmo, Hodgins, et al. 2022). RNs also described emotional strain linked to low confidence and systemic pressures, particularly in areas such as pain assessment and responding to distress, highlighting workforce well‐being as part of the wider care ecology (Aston et al. 2014b; Carter et al. 2016). Emotional intelligence training has been shown to enhance staff responsiveness and reduce stress, suggesting a pathway for strengthening both workforce well‐being and quality of care (Embregts et al. 2017; Zijlmans et al. 2015). Further research is needed to examine how relationships between individuals with severe intellectual disabilities, their families and HCPs can be better supported to meet core psychological needs across care settings (Kuld et al. 2023).

### 4.1 Review Strengths and Limitations

A key strength of this review lies in its novel and integrative scope, representing the first attempt to synthesise evidence across families, RNs, nursing students and nurse educators in relation to hospital care for children with intellectual disabilities. Importantly, the absence of studies addressing students' and educators' perspectives constitutes a substantive finding that exposes a critical gap in the literature, rather than a limitation of the review. In addition, the use of a robust mixed methods systematic review with a convergent integrated approach enabled the integration of qualitative and ‘qualitised’ findings, supporting a nuanced and holistic synthesis of relational, emotional and contextual dimensions of care that would not be achievable through single‐method designs alone.

These strengths should be interpreted alongside several limitations. The review protocol was not formally registered, which may limit transparency regarding potential post hoc modifications to review processes. The restriction to English‐language, peer‐reviewed studies and the predominance of evidence from high‐income countries, particularly the UK and Australia, may limit transferability to other contexts. In addition, the evidence base was heavily weighted towards inpatient hospital care, with relatively limited representation of outpatient services and inconsistent reporting of clinical settings. Consequently, it was not possible to determine whether the identified themes differed according to inpatient versus outpatient care or medical versus surgical contexts. Heterogeneity in study designs and variable reporting quality also constrained comparability and precluded meta‐analysis. Further limitations include inconsistent reporting of reflexivity, sampling and saturation, poor specification of intellectual disability levels and the continued under‐representation of direct child voice, with children's experiences often mediated through parents or professionals. This represents an important evidence gap, as the perspectives of children with intellectual disabilities are essential for informing inclusive and person‐centred hospital care (Wray et al. 2025).

### 4.2 Implications for Future Research

Future research should address three key gaps. First, there is a limited body of studies examining nursing students' and nurse educators' views and experiences of hospital care for children with intellectual disabilities, restricting understanding of how inclusive competence is developed within curricula and clinical learning environments. Second, greater priority should be given to the direct inclusion of children with intellectual disabilities through ethically robust, communication‐adapted methods, moving beyond predominant reliance on proxy accounts. Third, implementation‐focused research is needed to evaluate the real‐world impact of strategies such as hospital passports, identification systems and environmental adaptations, including their effects on safety, experience and staff confidence. Further studies from under‐represented regions, including the Middle East and other low‐ and middle‐income country (LMIC) contexts, are also required to support context‐sensitive and equitable advances in disability‐inclusive hospital care.

## 5 Conclusion

This review demonstrates that inequities in hospital care for children with intellectual disabilities are shaped by factors extending beyond individual encounters. Findings from families and RNs highlight how communication practices, recognition of family expertise, workforce preparedness and reasonable adjustments influence safety, emotional well‐being and equity. While some challenges reflect broader organisational and resource constraints, many improvements can be achieved through changes in everyday clinical practice. Evidence relating to nursing students and nurse educators was not identified within the included studies, highlighting an important gap in the current evidence base. The findings should also be interpreted in light of the predominance of evidence from high‐income countries, particularly the UK and Australia and may not fully reflect the realities of other healthcare systems, resource contexts or cultural settings. Further research from under‐represented regions is needed to strengthen the global evidence base and inform contextually appropriate approaches to disability‐inclusive hospital care.

## Funding

The authors have nothing to report.

## Ethics Statement

This study was a systematic review and did not require ethical approval.

## Conflicts of Interest

The authors declare no conflicts of interest.

## Supporting information


**Appendix A.** PRISMA checklist 2020.
**Appendix B.** Full search strategy.
**Appendix C.** Data extraction table.
**Appendix D.** Quality assessment of included studies using the MMAT.
**Appendix E.** Study countries and population.
**Appendix F.** Thematic findings.

## Data Availability

The data that support the findings of this study are available on request from the corresponding author. The data are not publicly available due to privacy or ethical restrictions.
