# Supplementary material for: The Views and Experiences of Families and Registered Nurses Regarding Hospital Care and Support of Children With Intellectual Disabilities: A Systematic Review
Source: J Appl Res Intellect Disabil. 2026 Aug 19;39(4):e70296. doi: 10.1111/jar.70296 (PMC13489723; doi:10.1111/jar.70296)
Supplement: Supplementary file 1 [file JAR-39-e70296-s001.docx]

**Supplementary** **Appendix A: PRISMA Checklist 2020**

| **Section and Topic** | **Item #** | **Checklist item** | **Location where item is reported** |
| --- | --- | --- | --- |
| **TITLE** | | |  |
| Title | 1 | Identify the report as a systematic review. | 1 |
| **ABSTRACT** | | |  |
| Abstract | 2 | See the PRISMA 2020 for Abstracts checklist. | 1 |
| **INTRODUCTION** | | |  |
| Rationale | 3 | Describe the rationale for the review in the context of existing knowledge. | 2–4 |
| Objectives | 4 | Provide an explicit statement of the objective(s) or question(s) the review addresses. | 4 |
| **METHODS** | | |  |
| Eligibility criteria | 5 | Specify the inclusion and exclusion criteria for the review and how studies were grouped for the syntheses. | 5-6  Table 1 |
| Information sources | 6 | Specify all databases, registers, websites, organisations, reference lists and other sources searched or consulted to identify studies. Specify the date when each source was last searched or consulted. | 7 |
| Search strategy | 7 | Present the full search strategies for all databases, registers and websites, including any filters and limits used. | 7-8  Table 2  Appendix B |
| Selection process | 8 | Specify the methods used to decide whether a study met the inclusion criteria of the review, including how many reviewers screened each record and each report retrieved, whether they worked independently, and if applicable, details of automation tools used in the process. | 8 |
| Data collection process | 9 | Specify the methods used to collect data from reports, including how many reviewers collected data from each report, whether they worked independently, any processes for obtaining or confirming data from study investigators, and if applicable, details of automation tools used in the process. | 9 |
| Data items | 10a | List and define all outcomes for which data were sought. Specify whether all results that were compatible with each outcome domain in each study were sought (e.g. for all measures, time points, analyses), and if not, the methods used to decide which results to collect. | 9 |
|  | 10b | List and define all other variables for which data were sought (e.g. participant and intervention characteristics, funding sources). Describe any assumptions made about any missing or unclear information. | 9 + 24 |
| Study risk of bias assessment | 11 | Specify the methods used to assess risk of bias in the included studies, including details of the tool(s) used, how many reviewers assessed each study and whether they worked independently, and if applicable, details of automation tools used in the process. | 8-9 |
| Effect measures | 12 | Specify for each outcome the effect measure(s) (e.g. risk ratio, mean difference) used in the synthesis or presentation of results. | NA |
| Synthesis methods | 13a | Describe the processes used to decide which studies were eligible for each synthesis (e.g. tabulating the study intervention characteristics and comparing against the planned groups for each synthesis (item #5)). | 9 -10  Appendix F |
|  | 13b | Describe any methods required to prepare the data for presentation or synthesis, such as handling of missing summary statistics, or data conversions. | 9-10 |
|  | 13c | Describe any methods used to tabulate or visually display results of individual studies and syntheses. | 9 |
|  | 13d | Describe any methods used to synthesize results and provide a rationale for the choice(s). If meta-analysis was performed, describe the model(s), method(s) to identify the presence and extent of statistical heterogeneity, and software package(s) used. | 9-10 |
|  | 13e | Describe any methods used to explore possible causes of heterogeneity among study results (e.g. subgroup analysis, meta-regression). | NA |
|  | 13f | Describe any sensitivity analyses conducted to assess robustness of the synthesized results. | NA |
| Reporting bias assessment | 14 | Describe any methods used to assess risk of bias due to missing results in a synthesis (arising from reporting biases). | NA |
| Certainty assessment | 15 | Describe any methods used to assess certainty (or confidence) in the body of evidence for an outcome. | NA |
| **RESULTS** | | |  |
| Study selection | 16a | Describe the results of the search and selection process, from the number of records identified in the search to the number of studies included in the review, ideally using a flow diagram. | 10 |
|  | 16b | Cite studies that might appear to meet the inclusion criteria, but which were excluded, and explain why they were excluded. | **11**  **Figure 1** |
| Study characteristics | 17 | Cite each included study and present its characteristics. | 12–22  Table 3 |
| Risk of bias in studies | 18 | Present assessments of risk of bias for each included study. | Appendix D |
| Results of individual studies | 19 | For all outcomes, present, for each study: (a) summary statistics for each group (where appropriate) and (b) an effect estimate and its precision (e.g. confidence/credible interval), ideally using structured tables or plots. | NA |
| Results of syntheses | 20a | For each synthesis, briefly summarise the characteristics and risk of bias among contributing studies. | 23-24 |
|  | 20b | Present results of all statistical syntheses conducted. If meta-analysis was done, present for each the summary estimate and its precision (e.g. confidence/credible interval) and measures of statistical heterogeneity. If comparing groups, describe the direction of the effect. | NA |
|  | 20c | Present results of all investigations of possible causes of heterogeneity among study results. | NA |
|  | 20d | Present results of all sensitivity analyses conducted to assess the robustness of the synthesized results. | NA |
| Reporting biases | 21 | Present assessments of risk of bias due to missing results (arising from reporting biases) for each synthesis assessed. | NA |
| Certainty of evidence | 22 | Present assessments of certainty (or confidence) in the body of evidence for each outcome assessed. | NA |
| **DISCUSSION** | | |  |
| Discussion | 23a | Provide a general interpretation of the results in the context of other evidence. | 32-37 |
|  | 23b | Discuss any limitations of the evidence included in the review. | 37-38 |
|  | 23c | Discuss any limitations of the review processes used. | 37-38 |
|  | 23d | Discuss implications of the results for practice, policy, and future research. | 32-39 |
| **OTHER INFORMATION** | | |  |
| Registration and protocol | 24a | Provide registration information for the review, including register name and registration number, or state that the review was not registered. | NA |
|  | 24b | Indicate where the review protocol can be accessed, or state that a protocol was not prepared. | NA |
|  | 24c | Describe and explain any amendments to information provided at registration or in the protocol. | NA |
| Support | 25 | Describe sources of financial or non-financial support for the review, and the role of the funders or sponsors in the review. | NA |
| Competing interests | 26 | Declare any competing interests of review authors. | Title page |
| Availability of data, code and other materials | 27 | Report which of the following are publicly available and where they can be found: template data collection forms; data extracted from included studies; data used for all analyses; analytic code; any other materials used in the review. | NA |

**Supplementary** **Appendix B: Full Search Strategy**

| **Database** | **No.** | **Index and Keywords** | **No. of Results** |
| --- | --- | --- | --- |
| CINAHL Complete | S1 | "nurs*" OR (MH "Parents") OR "famil*” OR "carer*" OR (MH "Caregivers") OR "nurs* educat*" | 1,556,923 |
|  | S2 | "Intellectual disab*" OR "Learning disab*" OR "developmental disab*" OR "mental deficiency" OR "mental* retard*" OR "intellectual developmental disorder" OR "intellectual developmental disability" OR (MH "Persons with Intellectual Disabilities") OR "cognitive disab*" OR "intellectual impairment" OR "mentally defective" OR "psychosocial retard*" | 54,700 |
|  | S3 | "child*" OR (MH "Young Adult") OR "young people" OR "adolescent*" OR "paediatric*" | 1,407,232 |
|  | S4 | (MH "Hospitals") OR (MH "Hospitals, Pediatric") OR (MH "Acute Care") OR (MH "Emergency Service") OR (MH "Emergency Room Visits") OR (MH "Patient Admission") OR (MH "Inpatients") OR "in-hospital" OR (MH "Hospitalization") OR (MH "Pediatric Care") OR (MH "Pediatric Units+") OR (MH "Child Health Services") OR ED | 426,770 |
|  | S5 | (S1 AND S2 AND S3 AND S4)  Limiter: Published Date ≤ 31 October 2025 | 504 |
|  | S6 | (S1 AND S2 AND S3 AND S4)  Limiter: English language | 498 |
| MEDLINE | #1 | nurs*.mp. OR parents/ OR Caregivers/ OR famil*.mp. OR carer*.mp. OR nurs* educat*.mp. | 2570092 |
|  | #2 | Intellectual disab*.mp. OR Developmental disab*.mp. OR learning disab*.mp. OR Intellectual developmental disability.mp. OR Intellectual developmental disorder.mp. OR Mental* retard*.mp. OR Mental deficiency.mp. OR Cognitive disab*.mp. OR Intellectual impairment.mp. OR mentally defective.mp. OR Psychosocial retard*.mp. | 146459 |
|  | #3 | child*.mp. OR young people.mp. OR Young Adult/ OR paediatric*.mp. OR adolescent*.mp. | 4940060 |
|  | #4 | hospitals/ OR hospitals, pediatric/ OR emergency departments.mp. OR Emergency Service, Hospital/ OR ED.mp. OR patient admission/ OR inpatients/ OR in-hospital.mp. OR hospitalization/ OR Child Health Services/ OR pediatric care.mp. OR pediatric units.mp. | 617349 |
|  | #5 | (#1 AND #2 AND #3 AND #4)  Limiter: Year (1860 – 2025) | 663 |
|  | #6 | (#1 AND #2 AND #3 AND #4)  Limiter: English language | 608 |
| APA PsycINFO | #1 | nurs*.mp. OR parents/ OR Caregivers/ OR famil*.mp. OR carer*.mp. OR nurs* educator*.mp. OR nurs* education.mp. | 760669 |
|  | #2 | Intellectual disab*.mp. OR Developmental disab*.mp. OR learning disab*.mp. OR Intellectual developmental disability.mp. OR Intellectual developmental disorder.mp. OR Mental* retard*.mp. OR Mental deficiency.mp. OR Cognitive disab*.mp. OR Intellectual impairment.mp. OR mentally defective.mp. OR Psychosocial retard*.mp. | 110124 |
|  | #3 | child*.mp. OR young people.mp. OR Young Adult/ OR paediatric*.mp. OR adolescent*.mp. | 1299173 |
|  | #4 | hospitals/ OR ED.mp. OR in-hospital.mp. OR hospitalization/ OR pediatric units.mp. OR exp Hospitalized Patients/ OR patient admission.mp. OR inpatient*.mp. OR hospitals, pediatric.mp. OR exp Emergency Services/ OR emergency departments.mp OR acute care.mp. OR Child Health Services.mp. OR pediatric care.mp. | 145354 |
|  | #5 | (#1 AND #2 AND #3 AND #4)  Limiter: Year (1860 – 2025) | 418 |
|  | #6 | (#1 AND #2 AND #3 AND #4)  Limiter: English language | 389 |
| Embase | #1 | nurs*.mp. OR parents/ OR Caregivers/ OR famil*.mp. OR carer*.mp. OR nurs* educat*.mp. | 3142535 |
|  | #2 | Intellectual disab*.mp. OR Developmental disab*.mp. OR learning disab*.mp. OR Intellectual developmental disability.mp. OR Intellectual developmental disorder.mp. OR Mental* retard*.mp. OR Mental deficiency.mp. OR Cognitive disab*.mp. OR Intellectual impairment.mp. OR mentally defective.mp. OR Psychosocial retard*.mp. | 165331 |
|  | #3 | child*.mp. OR young people.mp. OR Young Adult/ OR p?ediatric*.mp. OR adolescent*.mp. | 5190807 |
|  | #4 | hospitals/ OR hospitals, pediatric/ OR emergency departments.mp. OR Emergency Service, Hospital/ OR ED.mp. OR patient admission/ OR inpatients/ OR in-hospital.mp. OR hospitalization/ OR Child Health Services/ OR pediatric care.mp. OR pediatric units.mp. | 1881532 |
|  | #5 | (#1 AND #2 AND #3 AND #4)  Limiter: Year (1908 – 2025) | 1663 |
|  | #6 | (#1 AND #2 AND #3 AND #4)  Limiters: English language and article and journal | 741 |

**Supplementary** **Appendix C: Data Extraction Table**

| **NO** | **Study citation** | **Aim** | **Participants characteristics** | **Methods** | **Main findings** | **Recommendations** |
| --- | --- | --- | --- | --- | --- | --- |
| **1** | Aston et al. (2014a)  Canada | To gain deeper insight into how children with intellectual disabilities, their parents, and RNs experience and navigate the care process during their interactions throughout the child's hospital stay. | Mothers (n = 17), RNs (n = 12), and children (n = 8)  Children age range: mental age of 7 years or above.  Intellectual disability level: mild | Feminist poststructuralism; semi-structured interviews | Diagnoses supported access to care and understanding of needs but also reinforced negative stereotypes, creating tensions around roles and responsibilities. RNs reported anxiety and limited preparedness, relying on parents for guidance, which parents experienced as burdensome and reflective of inadequate communication. These dynamics highlighted the need for more respectful, effective communication. | The study recommended embedding intellectual disability-focused content within undergraduate healthcare curricula and promoting broader cultural change in societal and healthcare beliefs, values, and practices to support meaningful, sustained improvements in care. |
| **2** | Aston et al. (2014b)  Canada | To provide insight into the personal, institutional, and social experiences of children with intellectual disabilities, their parents, and the RNs who care for them within hospital settings. | Mothers (n = 17), RNs (n = 12), and children (n = 8)  Children age range: mental age of 7 years or above.  Intellectual disability level: mild | Feminist poststructuralism; semi-structured interviews | Relationships were central to positive hospital experiences and emotional well-being; yet time pressures, communication challenges, fear linked to limited knowledge and experience, stigma, and task-focused care undermined relationship-building, continuity of care, and responsiveness to emotional and psychological needs. | The study recommended critically challenging the medicalisation of hospital care, including underlying patriarchal assumptions, to support more equitable, relational, and family-centred approaches to caring for children with intellectual disabilities. |
| **3** | Avis and Reardon (2008)  UK | To explore parents' perspectives on the nursing care their child with additional needs and intellectual disabilities received during their hospital stay. | Parents (n = 12)  Children age range: under five, five, or older  Intellectual disability **level**: not clearly disclosed. | Qualitative; semi-structured interviews | Parents described an ongoing “hospital career” marked by cumulative emotional burden, with repeated encounters compounding distress. Trusting relationships with RNs were difficult to establish due to perceived judgement, ineffective communication, assumptions about parental expertise, and inconsistent empathy, negatively affecting trust and care quality. | The study recommended further research to examine factors influencing RNs’ confidence and skills in direct communication with children with intellectual disabilities, as well as their engagement with and responsiveness to parents’ needs. |
| **4** | Aydın et al. (2023)  Turkey | To gain a comprehensive understanding of the difficulties encountered by RNs in delivering care to children with intellectual disabilities in acute care environments. | RNs (n = 94) | Descriptive cross-sectional study; electronic surveys | Care was shaped by intersecting child-, family-, and system-level challenges, including communication difficulties, symptom interpretation, unpredictable behaviours, role conflicts, and limited family support. RNs reported limited preparedness due to insufficient knowledge and experience, while systemic constraints, such as high workloads, time pressures, unsuitable environments, lack of specialist support, and unclear legal guidance, further compromised care. | Enhanced education and training, development of specialised care guidelines, improved multidisciplinary collaboration, and addressing staffing levels and workload pressures to ensure high-quality care for children with intellectual disabilities. |
| **5** | Bates et al. (2020)  UK | To understand perceptions of specialist cleft services among children with clefts and intellectual disabilities, their parents, and HCPs, particularly regarding treatment, accessibility, and involvement in decision-making. | Children (n = 5), parents (n = 9), and HCPs (n = 9; RNs = 2)  Children age range: 10–16 years  Intellectual disability level: mild–moderate | Qualitative exploratory; semi‐structured interviews | Parents reported children’s distress during procedures, particularly needle use and unexpected restraint, with tensions arising from power imbalances, limited parental involvement, inadequate intellectual disability training, and inaccurate assumptions about children’s abilities. Recommended improvements included involving individuals with intellectual disabilities and families in training, alongside respectful communication, personalised care, and child-friendly environments. | Individualised treatment and communication tailored to the specific needs of children are essential, along with further research into the process of general anaesthetic induction for children with intellectual disabilities. |
| **6** | Breau et al. (2018)  Canada | To develop a more thorough understanding of the complex factors that can affect the hospital experiences of children with intellectual disabilities and their families. | Mothers (n = 17), RNs (n = 12), and children (n = 8)  Children age range: 3–19 years  Intellectual disability level: not clearly disclosed | Feminist poststructuralism; semi-structured interviews | RNs and parents identified inadequate formal education on caring for children with intellectual disabilities, leading to reliance on informal learning despite generally adequate physical care. Limited training contributed to low confidence, discomfort, fear, and communication challenges, while societal stigma and institutional practices de-emphasised relational care. Both groups emphasised the need for practical, family-centred training. | Additional research should be undertaken to investigate how power dynamics are negotiated in the context of caring for children with intellectual disabilities. |
| **7** | Brown and Guvenir (2009)  UK | To add to the growing body of data capturing the experiences of children with intellectual disabilities, as well as those of their families and RNs, during hospital admission. | Carers (n = 13), RNs (n = 13), and children (n = 2)  Children age range: 3–15 years  Intellectual disability level: not clearly disclosed | Qualitative; semi-structured interviews | Families and RNs reported heightened anxiety during hospital admissions, with families perceiving staff as underprepared even for planned care and remaining present to support their child due to limited training. RNs similarly described reliance on carers for behaviour management and communication. Both groups highlighted the value of individual rooms to reduce noise, stress, and anxiety. | Further research on the factors influencing hospital admission experiences is crucial for enhancing services and facilitating access to mainstream facilities and services for children with intellectual disabilities and their caregivers. |
| **8** | Carter et al. (2016)  UK | To understand how HCPs gain and build the knowledge and skills required to assess and manage pain in children with profound cognitive impairments. | HCPs (n = 19; RNs = 8) | Qualitative; semi-structured interviews | RNs reported difficulty assessing pain in children with cognitive impairments due to communication barriers, leading to stress and reliance on parents to interpret pain cues. Parental collaboration supported more individualised pain management, although experienced parents occasionally challenged clinical decisions, creating tension. | Mitigating the challenges associated with navigating uncertainty may require improved formal education and training opportunities for HCPs, aimed at addressing the ambiguity, complexity, and insecurity they currently encounter. |
| **9** | Cashin et al. (2022)  Australia | To compare Australian RNs' self-perceived levels of preparedness, knowledge, comfort, and confidence in caring for individuals with intellectual disabilities and/or autism spectrum disorder across various practice contexts, including primary care and community settings, intensive care units (ICUs), acute hospitals, emergency departments (EDs), paediatrics, and specialised intellectual disabilities/autism spectrum disorder services. | RNs (n = 639) | Cross-sectional descriptive study; electronic surveys | Paediatric RNs reported greater comfort in interacting with children with intellectual disabilities, discussing disabilities, building relationships, and managing behaviours compared with ICU and ED nurses. Despite this, they felt less prepared than intellectual disability specialists, identifying gaps in nursing curricula and advocating for targeted education and professional development to improve preparedness and confidence. | Further research is warranted that goes beyond self-reported knowledge to assess the actual knowledge levels of practicing RNs in their care for individuals with intellectual disability and/or autism spectrum disorder. |
| **10** | Dinsmore (2012)  UK | To assess qualitatively the overall hospital experiences of individuals with intellectual disabilities and their caregivers at National Health service (NHS) hospitals in Merseyside. | Carers (n = 10), adults with intellectual disability (n = 5)  Children age range: 8–51 years.  Intellectual disability level: moderate–profound | Qualitative; semi-structured interviews | The study identified gaps in specialist intellectual disability nursing provision, with carers’ contributions to care planning frequently overlooked. Deficits in communication, post-discharge support, and accessible health information were evident, while flexible care routines were highlighted as essential for minimising risk. | Hospitals should enhance awareness of intellectual disability liaison RNs, actively engage caregivers in patient care, provide clear post-hospital instructions, implement flexible routines, offer ongoing staff training on disabilities, and ensure that information is easily accessible. |
| **11** | Downs et al. (2016)  Australia | To assess family satisfaction following spinal fusion surgery in girls with Rett syndrome and to understand family explanations for what they found satisfying or dissatisfying in their clinical care experience. | Families (n = 392)  Age at data collection: median 21y 7m (range: 9y 6m–35y 1m)  Intellectual disability level**:** not clearly disclosed | A questionnaire with closed-ended and open-ended questions | Families reported high satisfaction with clinical care, particularly in surgical and ICU settings, attributing positive experiences to specialist expertise and effective communication that supported partnership. In contrast, inconsistent care, especially in post-operative pain management, and delays caused distress. Parents valued staff recognition of their insights, reinforcing the importance of clear communication and caregiver involvement. | The study recommended optimising surgical timing and expertise, strengthening clinician-family partnerships, ensuring consistent pain management across care settings, and providing ongoing long-term surveillance to support safe and effective care. |
| **12** | Iversen et al. (2013)  Norway | To understand the lived experiences of parents whose children with cerebral palsy are undergoing surgical procedures. | Parents (n = 12)  Children age range: 8–16 years  Intellectual disability level: not clearly disclosed | Hermeneutic phenomenology; in‐depth interviews | Parents experienced heightened vulnerability and emotional distress, particularly around procedures and staff understanding of their child’s needs. Trust in HCPs depended on recognition of parental expertise and effective communication, especially when children could not verbalise needs. Empathic, collaborative care was essential to address both child and parental emotional needs. | This study recommends providing ICU RNs with training focused on parents' perspectives and developing improved communication protocols to enhance collaboration, and care quality, and alleviate family distress during critical situations. |
| **13** | Jensen et al. (2025)  USA | To gain insight into what children with medical complexities  and their caregivers experience, examine how the COVID-19 pandemic affected them, and identify caregivers’ recommendations for strengthening the healthcare their children receive. | Caregivers (n = 175)  Children age range: five to twenty-one  Intellectual disability level: not clearly disclosed | An explanatory sequential mixed method study; surveys and in-depth interviews. | Children with intellectual disabilities required frequent and intensive hospital care, with repeated interventions contributing to anxiety, fear, and medical trauma, particularly for non-verbal or developmentally delayed children. Hospital encounters were emotionally demanding for families, who valued compassionate, collaborative HCPs and partnership in decision-making. Fragmented services and poor care coordination placed heavy advocacy burdens on caregivers, often leaving them feeling exhausted and undervalued despite their expertise. | The study recommended future research incorporating perspectives of HCPs, siblings, and, where possible, children with medical complexities, using validated measures, longitudinal designs, and more diverse samples, alongside examination of provider training, relational skills, and care delivery across clinical settings. |
| **14** | Kenten et al. (2019)  UK | To determine the methods utilised in English hospitals for identifying children with intellectual disabilities. | HCPs (n = 2326; 1020 RNs) | Structured interviews and  questionnaires | RNs valued identifying children with intellectual disabilities to support safe, individualised care but faced inconsistent practices and reliance on GP referrals, school information, and parental input. While many supported systematic “flagging,” concerns about stigma and family resistance were noted; RNs in hospitals with established flagging systems reported greater confidence. Communication was further hindered by language barriers, highlighting the need for additional intellectual disability-specific training. | Additional research is required to uncover the root causes, including the potential need to enhance the quantity and quality of undergraduate training related to intellectual disabilities. |
| **15** | Lamptey (2022)  Ghana | To explore the experiences of families as they navigate the Ghanaian health system to meet the general health needs of their children with intellectual and developmental disabilities. | Caregivers (n = 22)  Children age range: 3–18 years  Intellectual disability level: not clearly disclosed | Qualitative interpretive description; semi-structured interviews | Barriers to care included service denial, long waiting times, and negative HCP attitudes linked to limited flexibility in addressing children’s needs. Difficulties managing functional and behavioural challenges sometimes led to restraint or reliance on prior treatment approaches. Communication challenges often shifted interactions to families, while variability in infrastructure and resources resulted in inconsistent care coordination and fragmented services. | The study recommended further research into factors influencing HCPs’ decisions to provide expedited services for children with intellectual disabilities, alongside greater inclusion of fathers, single-parent families, and socioeconomically disadvantaged households to capture diverse care experiences and inequities. |
| **16** | Lewis et al. (2019)  Australia | To gain insight into RNs’ experiences of providing care for children with intellectual disabilities within an acute paediatric hospital environment. | RNs (n = 8) | Qualitative; one-on-one, face-to-face interviews | Care for children with intellectual disabilities required adaptations, with communication barriers and complex routines increasing nursing workload. Maintaining familiar routines was crucial for minimising distress and supporting effective care, while strong nurse-parent relationships, grounded in communication and collaboration, enabled more personalised care. | The study recommended future research exploring RNs’ experiences across care settings and hospital stays, with focus on the development of nurse-family relationships over time and inclusion of parents, partner caregivers, and children with intellectual disabilities where feasible. |
| **17** | Mimmo et al. (2022b)  Australia | To explore the factors that define a positive care experience for inpatient children with intellectual disabilities, as perceived by RNs. | RNs (n = 29) | Qualitative interpretive; focus groups | RNs described quality care as grounded in rapport-building, understanding children’s individual needs, and collaboration with parents. Effective communication, trust, and empathy underpinned strong relationships, with prior experience informing care. Time and resource constraints limited individualised attention, increasing reliance on parents, while paediatric RNs’ professional fulfilment supported care quality, including during the COVID-19 pandemic. | Health services should move beyond existing indicators of safe and quality care and consider utilising a conceptual model of safe care as a foundation for developing patient experiences and care partnerships for inpatient children with intellectual disabilities. |
| **18** | Moloney et al. (2023)  Ireland | To elucidate parents' perspectives on the reasonable adjustments made in acute settings for individuals with intellectual disabilities. | Parents (n = 6)  Children age range: 10–19 years  Intellectual disability level: mild, moderate or severe | Qualitative descriptive; in-depth semi-structured interviews | The lack of reasonable adjustments and person-centred planning undermined care experiences, contributing to distress for children with intellectual disabilities and their families. Parents highlighted the absence of sensory-friendly environments, shorter waiting times, and quiet spaces, and described ongoing advocacy efforts that led to feelings of isolation and being perceived as “pushy.” A more comprehensive approach incorporating person-centred care, improved communication, targeted training, and dedicated advocacy roles was identified as essential. | The study recommended shifting from exploratory to implementation-focused research to support practical application of reasonable adjustments, using frameworks centred on person-centred care, effective communication, education, and advocacy, with early and sustained patient and public involvement. |
| **19** | Murdoch and Chang (2022)  UK | To understand parents' experiences of caring for their child with intellectual disabilities and the procedural anxiety encountered in a hospital setting. | Parents (n = 6)  Children age range: 3–18 years  Intellectual disability level: not clearly disclosed | Interpretative phenomenological analysis (IPA); remote one-to-one semi-structured interviews | Parents experienced significant emotional strain, guilt, and distress, particularly during restraint procedures. Advocacy efforts elicited mixed responses, with some parents feeling dismissed while others gained confidence. Limited specialist support, inconsistent staff skills, and difficulties meeting children’s specific needs heightened anxiety and isolation, with pressures intensified during the COVID-19 pandemic. | Further research focused on identifying effective nursing strategies to improve parental experiences would be beneficial for enhancing care for this patient population. |
| **20** | Ong et al. (2024)  Australia | To examine patient safety concerns specific to children with intellectual disabilities. Additionally, to articulate these issues to inform modifications to the Australian Patient Safety Education Framework (APSEF) domains and subdomains for this demographic. | Parents (n = 13)  Children age range: 5–14 years  Intellectual disability level: not clearly disclosed | Qualitative; semi-structured interviews | Parents emphasised that safety encompassed emotional and psychological well-being and reported significant challenges arising from dismissive attitudes, poor communication, and limited access to tailored resources. They felt underutilised as safety advocates yet burdened with care responsibilities without adequate support, contributing to adverse outcomes. Effective care required personalised communication strategies and systemic adjustments, including improved coordination, sensory-friendly environments, accessible equipment, disability-specific training, and continuity of care. | The study recommended education programmes that prioritise competencies in engaging children with intellectual disabilities, delivering patient- and family-centred care, addressing systemic barriers, and providing reasonable adjustments within adapted care frameworks. |
| **21** | Oulton et al. (2015)  UK | To gain a deeper understanding of the perspectives of parents, clients, and staff regarding the needs of children with intellectual disabilities and their families during hospital stay. | HCPs (n = 27; 10 RNs) | Ethnographic study | Individualised care depended on adequate training and experience, clear identification of children with intellectual disabilities, recognition of child-specific needs, and attention to creating safe, familiar environments, supported by appropriate resources. Parents were central to enabling personalised care; nevertheless, gaps in staff knowledge, experience, and communication often resulted in over-reliance on parental input rather than genuine collaborative partnership. | Further research is necessary to explore the impact of this role on staff's capacity to provide individualised care to these patients. |
| **22** | Oulton et al. (2018)  UK | To enhance understanding of the needs and experiences of children with intellectual disabilities in the context of hospitalisation. | Children (n = 9) and at least one of their parents.  Children age range: 4–21 years  Intellectual disability level: varying degrees; specific levels not disclosed. | Ethnographic study | Children with intellectual disabilities valued small, personalised gestures and familiar routines, while unnecessary waiting and boredom contributed to stress. Maintaining comfort through meaningful activities and avoiding assumptions about abilities were essential, with tailored care prioritising communication, choice, and individual preferences to reduce anxiety and enhance hospital experiences. | It is essential for the authors to persist in questioning the notion that it is permissible to exclude children with intellectual disabilities from research due to perceived difficulties in their participation. |
| **23** | Oulton et al. (2019)  UK | To examine the organisational context of healthcare delivery for children with intellectual disabilities and to compare staff perceptions regarding their ability to identify and address the needs of those with and without intellectual disabilities.  To assess the nature and extent of dedicated intellectual disability nursing services in children’s hospitals and to contrast the views of staff working in hospitals with dedicated intellectual disability nurses with those in hospitals without such services. | HCPs (n = 1729; 850 RNs) | Semi-structured interviews and questionnaires | Limited intellectual disability nurse support was valued and enhanced RNs’ perceived capability but did not improve confidence, perceptions of patient value, safety, or access to care. Inconsistent policy understanding, shortages of senior specialist roles, and mixed organisational commitment constrained systemic progress. RNs struggled to implement proactive adjustments, particularly in emergencies, while persistent communication challenges, especially with non-verbal children, and underuse of health passports undermined care quality. Families continued to advocate for care, with past negative experiences shaping trust and generating complaints. | The study recommended clearer operationalisation of intellectual disability nurse roles within children’s hospitals, alongside robust monitoring, and evaluation mechanisms to maximise their impact for staff and families. |
| **24** | Oulton et al. (2020)  UK | To gain insight into the experiences of parents of children with intellectual disabilities in the hospital setting, as well as their expectations for their relationship with HCPs. | Parents (n = 9)  Children age range: 4–21 years  Intellectual disability level: varying degrees; specific levels not disclosed. | Ethnographic approach | Strong parent-HCP partnerships, grounded in trust, clear communication, and respect for parental expertise, were central to positive care experiences. Parents sought prepared, accessible professionals who provided consistent information and shared responsibility for care; when these elements were absent, families felt isolated and overburdened, underscoring the need for healthcare systems to support genuine, balanced partnerships. | Establishing trust in the system from the beginning is crucial for ensuring that children with intellectual disabilities and their families receive the appropriate support and care. |
| **25** | Oulton et al. (2022)  UK | To identify the cross-organisational, organisational, and individual factors that facilitate or obstruct equitable access to high-quality care and services for children with intellectual disabilities and their families within NHS hospitals, and to create guidance for NHS trusts based on these insights. | HCPs (n = 2853; 1109 RNs; number of RNs in specific phases was not specified)  **Children (n = 1419; 198 with intellectual disability)**  **Families (n = 1371; number of parents of children with intellectual disability was not specified)**  Children age range: 0–18 years  Intellectual disability level: not clearly disclosed | A four-phase transformative, mixed methods case study. | RNs reported gaps in dedicated intellectual disability policies, inconsistent access to specialist support, and limited sensory-friendly environments, alongside constraints in training, equipment, and family engagement that hindered individualised care. Parents similarly emphasised the need for personalised care due to children’s anxiety and communication challenges, citing inadequate staff expertise, poor supervision, insufficient environmental adjustments, and discharge delays that reduced preparedness and satisfaction. | The study recommended implementing reasonable adjustments to support individualised care, strengthening HCP training and experiential learning in intellectual disability care, and promoting pre-admission collaboration with parents to build knowledge of each child’s needs. |
| **26** | Oulton et al. (2024)  UK | To offer a comprehensive view of the emotional and physical effects of hospitalisation on children with intellectual disabilities, from both the children’s and parents’ perspectives. | Parents (n = 52) and children (n = 42)  Children age range: age not disclosed  Intellectual disability level: not clearly disclosed | A four-phase transformative, mixed methods case study. | Hospital care for children with intellectual disabilities involved interconnected challenges, including disruption of familiar routines, sensory overload, procedure-related anxiety, and difficulties with pain recognition and management due to communication barriers, all of which contributed to distress in unfamiliar environments. | It is essential to address the training needs of staff to ensure that care is tailored to the individual needs of each child. |
| **27** | Oulton et al. (2025)  UK | To explore parents', carers', and staff members'  perceptions of safety issues related to children with intellectual disabilities during inpatient stays in English  hospitals, utilising findings from [NAME OF STUDY]. | Parents (n = 52) and HCPs  (n = 98; number of RNs not specified)  Children age range: 6 years or over  Intellectual disability level: not clearly disclosed | Qualitative; semi-structured interviews | Parents described hospital care as safety-critical, requiring constant vigilance due to children’s limited safety awareness, communication difficulties, and anxiety-related behaviours, often compensating for gaps in supervision and variable staff expertise. RNs similarly identified heightened safety risks, particularly for non-verbal children, alongside challenges managing behaviours and delivering essential care in poorly adapted environments with limited staffing and training. Both groups emphasised the need for disability-specific education, safer environments, and collaborative partnerships to address persistent safety gaps. | Hospitals should adopt a tailored risk-assessment approach, strengthen staff training, improve environmental safety, and work in genuine partnership with parents to ensure safe, well-prepared, and equitable care for children with intellectual disabilities. |
| **28** | Seliner et al. (2016)  Switzerland | To evaluate the burden of care experienced by parents, their satisfaction with family-centred care, and the health-related quality of life (HRQoL) of both parents and their hospitalised children with profound intellectual and multiple disabilities, as well as to explore the relationships among these variables. | Mothers (n = 98) and fathers (n = 19)  Age range: 1–18 years  Intellectual disability level: profound | Cross-sectional study; surveys and qualitative questions | The findings underscored the need to strengthen family-centred care practices to reduce parental stress and support improved outcomes for hospitalised children and their families. | Future studies should avoid relying solely on children's HRQoL measures and consider supplementing parental reports with insights from other sources, such as RNs. |

**Supplementary** **Appendix D: Quality Assessment of Included Studies Using the MMAT**

| **Study citation** | **Qualitative** | | | | | **Quantitative descriptive** | | | | | **Mixed methods** | | | | |
| --- | --- | --- | --- | --- | --- | --- | --- | --- | --- | --- | --- | --- | --- | --- | --- |
|  | 1.1 | 1.2 | 1.3 | 1.4 | 1.5 | 4.1 | 4.2 | 4.3 | 4.4 | 4.5 | 5.1 | 5.2 | 5.3 | 5.4 | 5.5 |
| Aston et al. (2014a) | **Y** | **Y** | **Y** | **Y** | **Y** | **..** | **..** | **..** | **..** | **..** | **..** | **..** | **..** | **..** | **..** |
| Aston et al. (2014b) | **Y** | **Y** | **CT** | **Y** | **Y** | **..** | **..** | **..** | **..** | **..** | **..** | **..** | **..** | **..** | **..** |
| Avis and Reardon (2008) | **Y** | **Y** | **Y** | **Y** | **Y** | **..** | **..** | **..** | **..** | **..** | **..** | **..** | **..** | **..** | **..** |
| Aydın et al. (2023) | **..** | **..** | **..** | **..** | **..** | **Y** | **N** | **Y** | **CT** | **Y** | **..** | **..** | **..** | **..** | **..** |
| Bates et al. (2020) | **Y** | **Y** | **Y** | **Y** | **Y** | **..** | **..** | **..** | **..** | **..** | **..** | **..** | **..** | **..** | **..** |
| Breau et al. (2018) | **Y** | **Y** | **Y** | **Y** | **Y** | **..** | **..** | **..** | **..** | **..** | **..** | **..** | **..** | **..** | **..** |
| Brown and Guvenir (2009) | **CT** | **CT** | **Y** | **Y** | **Y** | **..** | **..** | **..** | **..** | **..** | **..** | **..** | **..** | **..** | **..** |
| Carter et al. (2016) | **Y** | **Y** | **Y** | **Y** | **Y** | **..** | **..** | **..** | **..** | **..** | **..** | **..** | **..** | **..** | **..** |
| Cashin et al. (2022) | **..** | **..** | **..** | **..** | **..** | **Y** | **Y** | **Y** | **CT** | **Y** | **..** | **..** | **..** | **..** | **..** |
| Dinsmore (2012) | **Y** | **Y** | **CT** | **Y** | **CT** | **..** | **..** | **..** | **..** | **..** | **..** | **..** | **..** | **..** | **..** |
| Downs et al. (2016) | **..** | **..** | **..** | **..** | **..** | **..** | **..** | **..** | **..** | **..** | **Y** | **Y** | **N** | **Y** | **CT** |
| Iversen et al. (2013) | **Y** | **Y** | **Y** | **Y** | **Y** | **..** | **..** | **..** | **..** | **..** | **..** | **..** | **..** | **..** | **..** |
| Jensen et al. (2025) | **..** | **..** | **..** | **..** | **..** | **..** | **..** | **..** | **..** | **..** | **Y** | **Y** | **Y** | **CT** | **Y** |
| Kenten et al. (2019) | **..** | **..** | **..** | **..** | **..** | **..** | **..** | **..** | **..** | **..** | **Y** | **Y** | **Y** | **Y** | **Y** |
| Lamptey (2022) | **Y** | **Y** | **Y** | **Y** | **Y** | **..** | **..** | **..** | **..** | **..** | **..** | **..** | **..** | **..** | **..** |
| Lewis et al. (2019) | **Y** | **CT** | **Y** | **Y** | **Y** | **..** | **..** | **..** | **..** | **..** | **..** | **..** | **..** | **..** | **..** |
| Mimmo et al. (2022b) | **Y** | **Y** | **Y** | **Y** | **Y** | **..** | **..** | **..** | **..** | **..** | **..** | **..** | **..** | **..** | **..** |
| Moloney et al. (2023) | **Y** | **Y** | **Y** | **Y** | **Y** | **..** | **..** | **..** | **..** | **..** | **..** | **..** | **..** | **..** | **..** |
| Murdoch and Chang (2022) | **Y** | **Y** | **Y** | **Y** | **Y** | **..** | **..** | **..** | **..** | **..** | **..** | **..** | **..** | **..** | **..** |
| Ong et al. (2024) | **Y** | **Y** | **Y** | **Y** | **Y** | **..** | **..** | **..** | **..** | **..** | **..** | **..** | **..** | **..** | **..** |
| Oulton et al. (2015) | **Y** | **Y** | **Y** | **Y** | **Y** | **..** | **..** | **..** | **..** | **..** | **..** | **..** | **..** | **..** | **..** |
| Oulton et al. (2018) | **Y** | **Y** | **Y** | **Y** | **Y** | **..** | **..** | **..** | **..** | **..** | **..** | **..** | **..** | **..** | **..** |
| Oulton et al. (2019) | **..** | **..** | **..** | **..** | **..** | **..** | **..** | **..** | **..** | **..** | **Y** | **Y** | **Y** | **Y** | **CT** |
| Oulton et al. (2020) | **Y** | **Y** | **Y** | **Y** | **Y** | **..** | **..** | **..** | **..** | **..** | **..** | **..** | **..** | **..** | **..** |
| Oulton et al. (2022) | **..** | **..** | **..** | **..** | **..** | **..** | **..** | **..** | **..** | **..** | **Y** | **Y** | **Y** | **Y** | **CT** |
| Oulton et al. (2024) | **..** | **..** | **..** | **..** | **..** | **..** | **..** | **..** | **..** | **..** | **Y** | **Y** | **Y** | **Y** | **CT** |
| Oulton et al. (2025) | **Y** | **Y** | **Y** | **Y** | **Y** | **..** | **..** | **..** | **..** | **..** | **..** | **..** | **..** | **..** | **..** |
| Seliner et al. (2016) | **..** | **..** | **..** | **..** | **..** | **..** | **..** | **..** | **..** | **..** | **Y** | **Y** | **CT** | **Y** | **N** |

What each number corresponds to 1·1, is the qualitative approach appropriate to answer the research question? 1·2, are the qualitative data collection methods adequate to address the research question? 1·3, are the findings adequately derived from the data? 1·4, is the interpretation of results sufficiently substantiated by data? 1·5, is there coherence between qualitative data sources, collection, analysis, and interpretation? 4·1, is the sampling strategy relevant to address the research question? 4·2, is the sample representative of the target population? 4·3, are the measurements appropriate? 4·4, is the risk of non-response bias low? 4·5, is the statistical analysis appropriate to answer the research question? 5·1, is there an adequate rationale for using a mixed methods design to address the research question? 5·2, are the different components of the study effectively integrated to answer the research question? 5·3, are the outputs of the integration of qualitative and quantitative components adequately interpreted? 5·4, are divergences and inconsistencies between quantitative and qualitative results adequately addressed? 5·5, do the different components of the study adhere to the quality criteria of each tradition of the methods involved? Y=yes. N=no. C=cannot tell.

Y= yes, indicates a clear statement appears in the paper which directly answers the question.

N = no, the answer indicating that the question has not been met in the paper.

CT = cannot tell, indicates there is no clear answer and/or adequate information to the question.

**Supplementary** **Appendix E: Study Countries and Population**

Countries

|  | Sales |
| --- | --- |
| United Kingdom | 14 |
| Austraila | 5 |
| Canada | 3 |
| Norway | 1 |
| United States | 1 |
| Ireland | 1 |
| Switzerland | 1 |
| Turkey | 1 |
| Ghana | 1 |

Participants number

|  | Sales |
| --- | --- |
| Families | 2184 |
| Children and adults | 1448 |
| Healthcare professionals | 3703 |

**Supplementary** **Appendix F:** **Thematic Findings**

|  | **The Significance of Communication** | **Partnership and Family Empowerment** | **Environmental and Systemic Barriers** | **The Role of Education and Professional Development** | **Emotional and Psychological Well-being** |
| --- | --- | --- | --- | --- | --- |
| **Aston et al. (2014a)** |  |  |  |  |  |
| **Aston et al. (2014b)** |  |  |  |  |  |
| **Avis and Reardon (2008)** |  |  |  |  |  |
| **Aydın et al. (2023)** |  |  |  |  |  |
| **Bates et al. (2020)** |  |  |  |  |  |
| **Breau et al. (2018)** |  |  |  |  |  |
| **Brown and Guvenir (2009)** |  |  |  |  |  |
| **Carter et al. (2016)** |  |  |  |  |  |
| **Cashin et al. (2022)** |  |  |  |  |  |
| **Dinsmore (2012)** |  |  |  |  |  |
| **Downs et al. (2016)** |  |  |  |  |  |
| **Iversen et al. (2013)** |  |  |  |  |  |
| **Jensen et al. (2025)** |  |  |  |  |  |
| **Kenten et al. (2019)** |  |  |  |  |  |
| **Lamptey (2022)** |  |  |  |  |  |
| **Lewis et al. (2019)** |  |  |  |  |  |
| **Mimmo et al. (2022b)** |  |  |  |  |  |
| **Moloney et al. (2023)** |  |  |  |  |  |
| **Murdoch and Chang (2022)** |  |  |  |  |  |
| **Ong et al. (2024)** |  |  |  |  |  |
| **Oulton et al. (2015)** |  |  |  |  |  |
| **Oulton et al. (2018)** |  |  |  |  |  |
| **Oulton et al. (2019)** |  |  |  |  |  |
| **Oulton et al. (2020)** |  |  |  |  |  |
| **Oulton et al. (2022)** |  |  |  |  |  |
| **Oulton et al. (2024)** |  |  |  |  |  |
| **Oulton et al. (2025)** |  |  |  |  |  |
| **Seliner et al. (2016)** |  |  |  |  |  |
